# Accounting for bias due to outcome data missing not at random: comparison and illustration of two approaches to probabilistic bias analysis: a simulation study

**DOI:** 10.1186/s12874-024-02382-4

**Published:** 2024-11-13

**Authors:** Emily Kawabata, Daniel Major-Smith, Gemma L. Clayton, Chin Yang Shapland, Tim P. Morris, Alice R. Carter, Alba Fernández-Sanlés, Maria Carolina Borges, Kate Tilling, Gareth J. Griffith, Louise A. C. Millard, George Davey Smith, Deborah A. Lawlor, Rachael A. Hughes

**Affiliations:** 1 MRC Integrative Epidemiology Unit, University of Bristol, Bristol, UK; 2 Population Health Sciences, Bristol Medical School, University of Bristol, Bristol, UK; 3 MRC Clinical Trials Unit at UCL, London, UK; 4 MRC Unit for Lifelong Health and Ageing at University College London, London, UK

**Keywords:** Bayesian bias analysis, Inverse probability weighting, Missing not at random, Monte Carlo bias analysis, Multiple imputation, Probabilistic bias analysis, Sensitivity analysis, UK Biobank

## Abstract

**Background:**

Bias from data missing not at random (MNAR) is a persistent concern in health-related research. A bias analysis quantitatively assesses how conclusions change under different assumptions about missingness using bias parameters that govern the magnitude and direction of the bias. Probabilistic bias analysis specifies a prior distribution for these parameters, explicitly incorporating available information and uncertainty about their true values. A Bayesian bias analysis combines the prior distribution with the data’s likelihood function whilst a Monte Carlo bias analysis samples the bias parameters directly from the prior distribution. No study has compared a Monte Carlo bias analysis to a Bayesian bias analysis in the context of MNAR missingness.

**Methods:**

We illustrate an accessible probabilistic bias analysis using the Monte Carlo bias analysis approach and a well-known imputation method. We designed a simulation study based on a motivating example from the UK Biobank study, where a large proportion of the outcome was missing and missingness was suspected to be MNAR. We compared the performance of our Monte Carlo bias analysis to a principled Bayesian bias analysis, complete case analysis (CCA) and multiple imputation (MI) assuming missing at random.

**Results:**

As expected, given the simulation study design, CCA and MI estimates were substantially biased, with 95% confidence interval coverages of 7–48%. Including auxiliary variables (i.e., variables not included in the substantive analysis that are predictive of missingness and the missing data) in MI’s imputation model amplified the bias due to assuming missing at random. With reasonably accurate and precise information about the bias parameter, the Monte Carlo bias analysis performed as well as the Bayesian bias analysis. However, when very limited information was provided about the bias parameter, only the Bayesian bias analysis was able to eliminate most of the bias due to MNAR whilst the Monte Carlo bias analysis performed no better than the CCA and MI.

**Conclusion:**

The Monte Carlo bias analysis we describe is easy to implement in standard software and, in the setting we explored, is a viable alternative to a Bayesian bias analysis. We caution careful consideration of choice of auxiliary variables when applying imputation where data may be MNAR.

**Supplementary Information:**

The online version contains supplementary material available at 10.1186/s12874-024-02382-4.

## Introduction

The main aim of many epidemiology studies is to estimate the causal effect of an exposure on an outcome (here onward, shortened to *exposure effect*). Inference about the exposure effect may be invalid when the sample included in the analysis is not a representative (random) sample of the target population. The choice of method for dealing with missing data partly depends on the mechanism causing the data to be missing (called *missingness mechanisms*). These mechanisms are commonly classified as missing completely at random (probability of missingness is independent of the observed and missing data), missing at random (MAR; probability of missingness is independent of the missing data given the observed data) and missing not at random (MNAR; probability of missingness depends on the missing data even after conditioning on the observed data) [1]. We focus on a MNAR missingness mechanism where the value of a variable directly affects its own probability of missingness [2]. Implementations of multiple imputation (MI) and inverse probability weighting (IPW) assume by default that data are MAR and so may give biased results when the missingness mechanism is MNAR. Note that implementations of MI and IPW incorporating MNAR mechanisms also exist (e.g., [3–5]).

Information about the missingness mechanism may be available from ancillary data such as instruments for missingness [6], record-linkage data [7, 8], and responsiveness data [9]. In the absence of such information, the analyst cannot distinguish between MAR and MNAR missingness mechanisms based on the observed data only [10]. Instead, the analyst must base their decision on expert knowledge or available literature. When MNAR missingness is suspected, a bias analysis (also known as a sensitivity analysis) is recommended to quantify the potential impact of MNAR missingness on their study conclusions [11–13].

A bias analysis for MNAR missingness (here onward, shortened to *bias analysis*) requires a model (known as a *bias model*) for the data and missingness mechanism. Two commonly used approaches are selection models and pattern-mixture models [11] (chapter 15, references therein). In the context of an outcome MNAR, the selection model usually consists of a model for the substantive analysis of interest and a model for the missingness mechanism that characterizes how missingness depends on the outcome. In contrast, the pattern-mixture model describes how the distribution of the outcome depends on missingness and may consist of a model for the substantive analysis that differs between participants with observed and missing outcome. Both types of model can be fitted using maximum likelihood, within a Bayesian framework or using multiple imputation [11, 14, 15].

Under MNAR both the selection and pattern-mixture models are unidentified models since the observed data does not provide any information about the parameters governing the dependency between the outcome and missingness (known as bias or sensitivity parameters). Setting the bias parameters to prespecified values enables estimation of the remaining parameters of the model and provides an estimate of the exposure effect adjusted for bias due to MNAR (here onward, called the *bias-adjusted exposure effect estimate*). By changing the values of these bias parameters, a bias analysis estimates the bias-adjusted exposure effect under different assumptions about the missingness mechanism.

A bias analysis can be implemented as a deterministic or probabilistic bias analysis [12, 15]. In a deterministic bias analysis, a range of values is specified for all bias parameters and then for each plausible combination of values, the bias model is estimated by fixing the bias parameters to these values. This approach provides the analyst with information about the range of possible estimates for the exposure effect but does not indicate which of these estimates are most likely to occur, making interpretation of the results challenging [12]. Alternatively, a probabilistic bias analysis specifies a prior probability distribution for the bias parameters which explicitly incorporates the analyst’s assumptions about plausible values and the combinations of values most likely to occur. The probabilistic bias analysis generates a distribution of bias-adjusted exposure effect estimates which is then summarised as a point estimate (e.g., the median as a measure of central tendency) and a 95% interval estimate (e.g., 2.5th and 97.5th percentiles as limits of the interval) that accounts for the analyst’s uncertainty about the MNAR missingness mechanism in addition to the usual random sampling error.

A probabilistic bias analysis can be implemented as a Bayesian bias analysis (where the prior distribution of the bias parameters is combined with the likelihood function for the data) or as a Monte Carlo bias analysis (where values of the bias parameters are directly sampled from their prior distribution and then used to fix the bias parameters to enable estimation of the bias-adjusted exposure effect) [16]. Generally, a Monte Carlo bias analysis is simpler to understand, quicker and easier to implement as it requires no Bayesian computation [12, 17, 18]. We note that the term “Monte Carlo” is also used to describe simulation-based techniques for Bayesian inference. To avoid confusion, we shall use the term “Markov Chain Monte Carlo (MCMC)” when referring to sampling from a posterior distribution and “Monte Carlo bias analysis” when referring to a type of probabilistic bias analysis.

In the context of bias analysis to unmeasured confounding or misclassification, a small number of studies have compared a Monte Carlo bias analysis to a Bayesian bias analysis [16–21]. Along with some theoretical arguments, these studies indicate that the Monte Carlo bias analysis is a good approximation of a Bayesian bias analysis provided the prior distribution for the bias parameters only specifies plausible values given the observed data [17–20]. Otherwise, the Monte Carlo bias analysis can give interval estimates that are either too wide or too narrow [16, 19]. No study has compared a Monte Carlo bias analysis to a Bayesian bias analysis in the context of a bias analysis to MNAR missingness.

Currently, there is limited guidance on implementing a probabilistic bias analysis to data MNAR. Recent exceptions for cross-sectional analyses include: (1) a pattern-mixture approach where draws from a prior distribution (of the bias parameters) are used to impute a categorical covariate under MNAR [22, 23] and (2) a Bayesian implementation of a selection model for a partially observed continuous outcome [24]. Additionally, in the context of selection bias due to non-random selection of participants into a study, Banack et al. review and compare a Monte Carlo bias analysis to an alternative approach that simulates the entire dataset under different assumptions about the selection bias [25] and Jayaweera et al. conducted a Monte Carlo bias analysis by inversely weighting participants based on their probability of inclusion (i.e., participating and remaining in the study combined) [26].

In this paper, we illustrate a Monte Carlo bias analysis [12, 17] using a pattern-mixture version of fully conditional specification (FCS) imputation [5, 27, 28]. Via a data example and simulations, we compare the performance of our Monte Carlo bias analysis to a Bayesian bias analysis in a setting where a large proportion of the outcome is missing and missingness is suspected to be MNAR. R and Stata software code implementing the Monte Carlo and Bayesian bias analyses is available from https://github.com/MRCIEU/COVIDITY_ProbQBA.

## Methods

### Hypothetical example

We want to estimate the effect of an exposure (or treatment) $X$ on an outcome $Y$, denoted ${\beta }_{X}$. To estimate ${\beta }_{X}$, our substantive analysis is a generalised linear regression of $Y$ on $X$ adjusted for measured confounders $Z$ and $W$

1 $$E\left(Y|X,Z,W\right)={g}_{Y}^{-1}\left({\beta }_{0}+{\beta }_{X}X+{\beta }_{Z}Z+{\beta }_{W}W\right)$$

where ${g}_{Y}^{-1}\left(\cdot \right)$ denotes the inverse link function. We assume all confounders of the $Y$–$X$ association are measured and without error, and in the absence of missing data that the substantive analysis would give unbiased results for ${\beta }_{X}$. Outcome $Y$ is observed in a small proportion of study participants. The study recorded data on auxiliary variables (i.e., variables not included in the substantive analysis) that are predictive of the missing values of $Y$ and whether $Y$ was observed or missing. Also, a small proportion of participants are missing data on exposure $X$ and some of the confounders and auxiliary variables. Let $Z$ and $W$ denote the fully and partially observed confounders, respectively, and $A$ and $D$ denote the fully and partially observed auxiliary variables, respectively. To simplify the notation, and without loss of generality, we assume that $A$ denotes a single variable, and similarly for $D, Z$ and $W$. Binary variables ${M}^{Y},{M}^{X},{M}^{W}$ and ${M}^{D}$ denote the missingness indicators of $Y,X,W$ and $D$, respectively (e.g., ${M}^{Y}=1$ when $Y$ is missing and ${M}^{Y}=0$ otherwise).

**Fig. 1:**
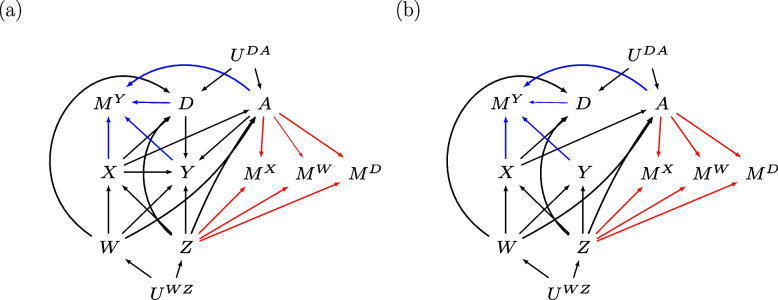
Missingness directed acyclic graphs (m-DAGs) of the scenario investigated by the simulation study when the exposure effect, ${\beta }_{X}$, is (**a**) not-null and (**b**) null. Black edges depict the relationships in the fully observed data, and the blue and red edges depict the missingness mechanisms of the outcome and baseline variables (exposure, confounders, and auxiliary variables), respectively

Figure 1 depicts two missingness directed acyclic graphs (m-DAGs [29]) showing the relationships among the variables of our substantive analysis of interest ($Y,X,Z$ and $W$), the auxiliary variables ($A$ and $D$), and the missingness mechanisms of $Y$, and of $X,W$ and $D$. Note that m-DAGs do not specify the form of these relationships (e.g., nonlinear relationships between variables). Exposure effect, ${\beta }_{X}$, represents the total effect of $X$ on $Y$ (i.e., direct effect and indirect effect via auxiliaries A and D). We consider two scenarios, when ${\beta }_{X}$ is not-null (Fig. 1a) and null (Fig. 1b). Note that ${U}^{WZ}$ and ${U}^{DA}$ denote unmeasured shared ancestors of $W$ and $Z$, and $D$ and $A$, respectively. Outcome $Y$ is MNAR depending on fully observed auxiliary $A$, the missing values of $Y$, and the observed and missing values of exposure $X$ and auxiliary $D$. Note that missingness of $Y$ does not depend on $W$ or $Z$, and we exclude the special case where the MNAR mechanism depends on $X$ and $Y$ independently [30]. Variables $X,W$ and $D$ are MAR depending on fully observed confounder $Z$ and auxiliary $A$; hence this MAR mechanism applies across all missing data patterns of $Y,X,W$, and $D$.

### Complete case analysis and ignorable missing data methods

Popular missing data methods include complete case analysis (CCA) and ignorable implementations of MI and IPW that assume MAR (hereafter referred to as MI and IPW, respectively). A detailed comparison of these methods is provided elsewhere (e.g., [10, 31, 32]).

For our hypothetical example, CCA and MI are expected to give a biased estimate for ${\beta }_{X}$ in both the null and not-null scenarios. Since missingness of $Y$ depends jointly on $X$ and $Y$, CCA is an invalid approach even when the substantive analysis is a logistic regression [30]. For MI, the MAR assumption is not valid, regardless of the variables included in the imputation model, since missingness of $Y$ depends directly on $Y$ (i.e., path $Y\to {M}^{Y}$). See the Supplementary materials for information about IPW in the context of our hypothetical example.

We next describe two non-ignorable missing data methods, a Bayesian bias analysis using a selection model (here onward, called *Bayesian SM*) and a Monte Carlo bias analysis using a pattern-mixture model (here onward, called *Monte Carlo NARFCS*). The bias models of Bayesian SM and Monte Carlo NARFCS consist of a collection of generalised linear regressions. For simplicity and without loss of generality, we describe Bayesian SM and Monte Carlo NARFCS with respect to continuous variable $X$ and binary variables $Y,W,A$ and $D$, whilst $Z$ is left unspecified and, by definition, missingness indicators ${M}^{Y},{M}^{X},{M}^{W}$ and ${M}^{D}$ are binary.

### Bayesian SM

#### Bias model specified as a selection model

We use the sequential modelling approach [33–36] to jointly model the substantive analysis, the MNAR missingness mechanism for $Y$, and the models to estimate the missing values of $X,W$ and $D$. The sequential modelling approach factorises a joint distribution into a sequence of simpler univariate distributions, where each univariate distribution is modelled using an appropriate regression model (e.g., linear regression for continuous variables and logistic regression for binary variables). We specify the following regression models for the joint distribution of $W,X,Y,A,D,{M}^{Y}\left|Z\right.$:

2 $$\begin{array}{l}Pr\left(W=1|Z\right)\sim expit\left\{{\eta}_{0}+{\eta}_{Z}Z\right\}, X\left|W,Z\right.\sim N\left({\zeta}_{0}+{\zeta}_{W}W+{\zeta}_{Z}Z,{\xi}^{2}\right),\\Pr\left(Y=1|X,W,Z\right)\sim expit\left\{{\beta}_{0}+{\beta}_{X}X+{\beta}_{W}W+{\beta}_{Z}Z\right\},\\Pr\left(A=1|Y,X,W,Z\right)\sim expit\left\{{\theta}_{0}+{\theta}_{Y}Y+{\theta}_{X}X+{\theta}_{W}W+{\theta}_{Z}Z\right\},\\Pr\left(D=1|A,Y,X,W,Z\right)\sim expit\left\{{\omega}_{0}+{\omega}_{A}A+{\omega}_{Y}Y+{\omega}_{X}X+{\omega}_{W}W+{\omega}_{Z}Z\right\},\\Pr\left({M}^{Y}=1|D,A,Y,X\right)\sim expit\left\{{\psi}_{0}+{\psi}_{D}D+{\psi}_{A}A+{\delta}^{SM}Y+{\psi}_{X}X\right\},\end{array}$$

where $expit\left\{k\right\}=exp\left\{k\right\}/\left(1+exp\left\{k\right\}\right)$, and ${\delta }^{SM}$ is the bias parameter representing the difference in the log-odds of observing $Y$ between those with $Y=1$ and $Y=0$, conditional on $D,A,Y$ and $X$. Note that as missingness of $\text{Y}$ is conditionally independent of $\text{W}$ and $\text{Z}$ given $\text{D},\text{A},\text{Y}$ and $\text{X}$ then the bias model correctly assumes that $\text{Pr}\left({\text{M}}^{\text{Y}}=1|\text{D},\text{A},\text{Y},\text{X},\text{W},\text{Z}\right)=\text{Pr}\left({\text{M}}^{\text{Y}}=1|\text{D},\text{A},\text{Y},\text{X}\right)$ for all values of $\text{W}$ and $\text{Z}$. Let ${\Psi }^{SM}$ denote the set of all estimable parameters of model [2] (i.e., all except ${\delta }^{SM}$), noting ${\Psi }^{SM}$ includes exposure effect ${\beta }_{X}$.

Different orderings of these regression models may result in different joint distributions [37]. We specified this ordering because it includes: the substantive analysis, a model for the MNAR missingness mechanism of $Y$, and incorporates auxiliary variables $A$ and $D$ without altering the substantive analysis. This ordering is compatible with a selection model framework. The ordering of the remaining models can be with respect to the amount of missing data (i.e., starting with the model for the variable with the least amount of missing data). Note that previous studies have reported that a Bayesian implementation of the sequential modelling approach appears robust to the ordering of the models [38, 39] but it may affect computational time [33].

#### Prior probability distributions

We assign independent prior distributions for all parameters. Following standard practice, we assign a normal distribution for each coefficient of the regression models and an inverse gamma distribution for the variance parameter of a linear regression [40]. For ${\delta }^{SM}$ we assign Normal distribution ${\delta }^{SM}\sim N\left({\mu }^{SM},{\sigma }^{SM}\right)$ where values for mean ${\mu }^{SM}$ and variance ${\sigma }^{SM}$ are chosen based on external information such as published results, expert opinion, or external data. In practice, external information about ${\delta }^{SM}$ may be unattainable. Instead, it may be easier to obtain external information about a related parameter (such as the marginal difference in the log-odds of observing $Y$ between those with $Y=1$ and $Y=0$) that can then be converted into information about ${\delta }^{SM}$. We illustrate this concept when deriving values for hyperparameters ${\mu }^{SM}$ and ${\sigma }^{SM}$ in our motivating example. For all remaining parameters, ${\Psi }^{SM}$, we assign vague priors; namely, $N\left(\text{0,100}\right)$ for the coefficients and Inv-Gamma(0.01,0.01) for the variances.

#### Bayesian implementation

In the Bayesian framework, Bayes’ theorem is applied to combine the prior distributions for the bias model parameters with the likelihood function for the data to obtain the joint posterior distribution of (${\delta }^{SM}, {\Psi }^{SM}$). Therefore, application of Bayes’ theorem may rule out certain values of ${\delta }^{SM}$ because they are incompatible with the data [16]. From the joint posterior distribution of (${\delta }^{SM}, {\Psi }^{SM}$), we can derive the conditional posterior distribution of a single parameter, such as ${\beta }_{X}$.

The Bayesian framework views the missing data of $Y,X,W,$ and $D$ and parameters ${\delta }^{SM}$ and ${\Psi }^{SM}$ as unknown quantities to be estimated. Since direct sampling from the joint distribution of these unknown quantities is difficult, we fit the selection model using MCMC estimation, specifically Gibbs sampling implemented by JAGS (version 4.3.0) [41–43] using R package *jagsUI* (version 1.5.2) [44].

### Monte Carlo NARFCS

#### Bias model specified as a pattern-mixture model

We use the Not-At-Random Fully Conditional Specification (NARFCS) approach [5] which is an MNAR extension of the MAR imputation method FCS [28] (see references therein for other variants of FCS). Like FCS, NARFCS imputes each variable under a separate univariate regression model (of type appropriate to the variable being imputed) and updates the missing data for each variable in turn using an iterative algorithm which we shall call the FCS algorithm [45, 46]. Note that the univariate distributions implied by these regression models may not be consistent with the same joint distribution and different orderings of these regression models within the FCS algorithm could lead to sampling from different joint distributions [46, 47]. In practice, FCS has been shown to be a robust approach even when the set of regression models are not compatible with the same joint distribution ( [46], references therein). The order in which the partially observed variables are updated within the FCS algorithm is typically determined by the amount of missing data [27].

We specify the following regression models for our NARFCS bias model:

3 $$\begin{array}{l}Pr\left(W=1|Y,X,D,A,Z,{M}^{Y},{M}^{X},{M}^{D}\right)\sim expit\left\{{\alpha }_{0}+{\alpha }_{Y}Y+{\alpha }_{X}X+{\alpha }_{D}D+{\alpha }_{A}A+{\alpha }_{Z}Z+{\alpha }_{{M}^{Y}}{M}^{Y}+{\alpha }_{{M}^{X}}{M}^{X}+{\alpha }_{{M}^{D}}{M}^{D}\right\},\\X\left|Y,W,D,A,Z,{M}^{Y},{M}^{W},{M}^{D}\right.\sim N\left({\gamma }_{0}+{\gamma }_{Y}Y+{\gamma }_{W}W+{\gamma }_{D}D+{\gamma }_{A}A+{\gamma }_{Z}Z+{\gamma }_{{M}^{Y}}{M}^{Y}+ {\gamma }_{{M}^{W}}{M}^{W}+{\gamma }_{{M}^{D}}{M}^{D},{\varepsilon }^{2}\right),\\Pr\left(D=1|Y,X,W,A,Z,{M}^{Y},{M}^{X},{M}^{W}\right)\sim expit\left\{{\kappa }_{0}+{\kappa }_{Y}Y+{\kappa }_{X}X+{\kappa }_{W}W+{\kappa }_{A}A+{\kappa }_{Z}Z+{\kappa }_{{M}^{Y}}{M}^{Y}+{\kappa }_{{M}^{X}}{M}^{X}+{\kappa }_{{M}^{W}}{M}^{W}\right\},\\Pr\left(Y=1|X,W,D,A,Z,{M}^{Y},{M}^{X},{M}^{W},{M}^{D}\right)\sim expit\left\{{\lambda }_{0}+{\lambda }_{X}X+{\lambda }_{W}W+{\lambda }_{D}D+{\lambda }_{A}A+{\lambda }_{Z}Z+{\delta }^{NARFCS}{M}^{Y}+{\lambda }_{{M}^{X}}{M}^{X}+{\lambda }_{{M}^{W}}{M}^{W}+{\lambda }_{{M}^{D}}{M}^{D}\right\}\end{array}$$

where ${\delta }^{NARFCS}$ is the bias parameter, representing the difference in the log-odds of $Y=1$ between those with observed and missing values of $Y$. Let ${\Psi }^{NARFCS}$ denote the set of all estimable parameters of model [3] (i.e., all except ${\delta }^{NARFCS}$), which does not include ${\beta }_{X}$.

NARFCS differs from FCS in two ways which we shall illustrate using the regression model for $Y$ in the bias model, [3], above. First, NARFCS includes missingness indicator ${M}^{Y}$ as an independent variable in the regression model for $Y$ in order to quantify how the distribution of $Y$ differs between participants with observed and missing values of $Y$. Hence NARFCS belongs to the class of pattern-mixture models. Second, NARFCS includes the missingness indicators of the other partially observed variables, ${M}^{X},{M}^{W},{M}^{D}$, as independent variables in the regression model for $Y$ in order to maximise the amount of correlation between the variables captured by the model [5]. Note that the regression model for $X$ omits ${M}^{X}$ as an independent variable because we assume $X$ is MAR given $A$ and $Z$. Similarly, for the regression models of $W$ and $D$.

#### Prior probability distributions

NARFCS does not assign a prior distribution for ${\delta }^{NARFCS},$ instead it fixes ${\delta }^{NARFCS}$ to a prespecified value before applying the FCS algorithm. For remaining parameters, ${\Psi }^{NARFCS}$, NARFCS independently samples the parameters of each regression model from a posterior distribution (or an approximation) under a vague prior distribution (to ensure uncertainty from estimating the imputation model parameters is propagated through to the resulting imputations [27]). For example, for a regression with coefficients $\upsilon$ (and if applicable variance parameter $\varsigma$) Stata command *mi impute chained* (version 17 [48]) and R package *mice* (version 3.14.0) specify prior $p\left(\nu ,\varsigma \right)\propto {}^{1}\!\left/ \!{}_{\varsigma }\right.$ for a linear regression and prior $p\left(\nu \right)\propto 1$ for a logistic regression [49, 50].

Note that Tompsett et al. [5] illustrate a deterministic bias analysis using NARFCS where (in the context of our hypothetical example) the user prespecifies multiple values for ${\delta }^{NARFCS}$ and then repeatedly applies NARFCS by fixing ${\delta }^{NARFCS}$ to each pre-specified value in turn. As we are implementing a probabilistic bias analysis using NARFCS, we must specify a prior distribution for ${\delta }^{NARFCS}$. In keeping with Bayesian SM, we use prior $p\left({\delta }^{NARFCS}\right)\sim N\left({\mu }^{NARFCS},{\sigma }^{NARFCS}\right)$ with ${\mu }^{NARFCS}$ and ${\sigma }^{NARFCS}$ set to values based on external information about ${\updelta }^{\text{NARFCS}}$, or more practically on a related parameter that is then converted into information about ${\updelta }^{\text{NARFCS}}$.

#### Monte Carlo bias analysis

The Monte Carlo bias analysis repeatedly samples directly from the prior distribution for ${\delta }^{NARFCS}$ before fitting the bias model. Therefore, no sampled values of ${\delta }^{NARFCS}$ are rejected due to incompatibility with the observed data. Using the NARFCS bias model, we generate a Monte Carlo frequency distribution of bias-adjusted estimates of ${\beta }_{X}$ by repeatedly carrying out the following steps $S$ $\left(S>1\right)$ times: for $s=1,\cdots ,S$

i. Randomly draw a value for the bias parameter directly from its prior distribution, ${\delta }^{NARFCS(s)}\sim N\left({\mu }^{NARFCS}, {\sigma }^{NARFCS}\right)$.
ii. Impute the observed data $K$ $\left(K\ge 1\right)$ times using the NARFCS bias model with the bias parameter fixed at ${\delta }^{NARFCS\left(s\right)}$. Fit the substantive analysis separately to each imputed dataset using maximum likelihood estimation and combine the multiple sets of results for ${\beta }_{X}$ using Rubin’s rules [1]. Let ${\widetilde{\beta }}_{X}^{{\delta }^{NARFCS(s)}}$ and ${\widetilde{V}}_{X}^{{\delta }^{NARFCS(s)}}$ denote the combined estimate of ${\beta }_{X}$ and accompanying variance, respectively.
iii. Incorporate random sampling error ${\widehat{\beta }}_{X}^{{\delta }^{NARFCS(s)}}\sim N\left({\widetilde{\beta }}_{X}^{{\delta }^{NARFCS(s)}},{\widetilde{V}}_{X}^{{\delta }^{NARFCS(s)}}\right)$.

After $S$ steps, we compute the median, 2.5th and 97.5th percentiles of the frequency distribution of ${\widehat{\beta }}_{X}^{{\delta }^{NARFCS(1)}},{\widehat{\beta }}_{X}^{{\delta }^{NARFCS(2)}},\cdots ,{\widehat{\beta }}_{X}^{{\delta }^{NARFCS(S)}}$ to obtain our Monte Carlo NARFCS point and interval estimates of ${\beta }_{X}$. Monte Carlo NARFCS was implemented in R using the NARFCS extension to *mice* [51] and in Stata using *mi impute* with option *offset*.

### Simulation study design

We compared the performance of Monte Carlo NARFCS with Bayesian SM when a large proportion of data were missing under a very strong MNAR mechanism. We evaluated these methods when the prior distribution for the bias parameter was (i) inaccurate and imprecise, (ii) accurate and reasonably precise, and (iii) accurate and very precise. We repeated the simulation study for ${\beta }_{X}=0$ and ${\beta }_{X}=\text{ln}(3)$ and for two data generating models: based on the selection model framework (SM data generating model) and the pattern-mixture model framework (PMM data generating model). For all combinations of the simulation settings, we generated 1000 simulated data sets, each with 100,000 observations for the full sample.

#### Generation of the complete data

The simulation study was based on the hypothetical example described above with the exception that $Z=\left({Z}_{1},{Z}_{2},{Z}_{3}\right)$ denotes three fully observed confounders and $A=\left({A}_{1},{A}_{2}\right)$ denotes two fully observed auxiliary variables. Exposure $X$ and ${Z}_{2}$ were continuous variables with mean 0 and standard deviation of 1, and the remaining variables were binary (${Z}_{1},{Z}_{3},A,$ outcome $Y$, partially observed confounder $W$, partially observed auxiliary $D$ and missingness indicators ${M}^{Y},{M}^{X},{M}^{W}$, and ${M}^{D}$).

First, we simulated (complete) data on $X,Y,Z,W,A,D,$ and ${M}^{Y}$ from their joint distribution factorised into a series univariate regressions: logistic regression for $Y,{Z}_{1},{Z}_{3},W,A,D,$ and ${M}^{Y}$, and linear regression for $X$ and ${Z}_{2}$. We considered two factorisations of this joint distribution, with the factorisation for the SM and PMM data generating models chosen to resemble the bias model of Bayesian SM and Monte Carlo NARFCS, respectively. See the Supplementary materials for further details.

Most of the parameter values of the SM data generating model were set to estimates from an analysis of a real dataset (our motivating example, described in the next section). Note that the value of ${\delta }^{SM}$ was artificially derived to simulate a very strong MNAR mechanism. The marginal prevalence of $Y$ was fixed at 5% for both the ${\beta }_{X}=0$ and ${\beta }_{X}=\text{ln}(3)$ scenarios. We were unable to analytically derive the corresponding parameter values of the PMM data generating model. Instead, we fitted the PMM data generating model to a dataset of 50,000,000 observations simulated under the SM data generating model and then used the resulting estimates as the parameter values of the PMM data generating model [4].

#### Generation of the missing data

Following generation of the complete data, which included missingness indicator ${M}^{Y}$, values of $Y$ were set to missing when ${M}^{Y}=1$. Missing data for $X,W,$ and $D$ were subsequently generated independently of each other and of $Y$ using the following missingness mechanisms of MAR given fully observed variables:

4 $$\begin{aligned} Pr\left({M}^{X}=1|{Z}_{1},{Z}_{2},{Z}_{3},{A}_{1},{A}_{2}\right)=expit\left\{-3.20+ 0.233\times {Z}_{1}-0.0570\times {Z}_{2}-0.133\times {Z}_{3}+ 0.363\times {A}_{1}+0.763\times {A}_{2}\right\},\\Pr\left({M}^{W}=1|{Z}_{1},{Z}_{2},{Z}_{3},{A}_{1},{A}_{2}\right)=expit\left\{-2.90+ 0.0720\times {Z}_{1}-0.232\times {Z}_{2}-0.774\times {Z}_{3}+ 0.169\times {A}_{1}+0.417\times {A}_{2}\right\},\\Pr\left({M}^{D}=1|{Z}_{1},{Z}_{2},{Z}_{3},{A}_{1},{A}_{2}\right)=expit\left\{-2.95- 0.0590\times {Z}_{1}-0.0290\times {Z}_{2}-0.190\times {Z}_{3}+ 0.130\times {A}_{1}+0.192\times {A}_{2}\right\}\end{aligned}$$

where all parameter values were derived from the observed data of our motivating example. These missingness mechanisms were the same for both the $\beta_{x}=0$ and $\beta_{x}=\text{ln}(3)$ scenarios and the SM and PMM data generating models, resulting in a non-monotone missingness pattern. Close to 5% of the observations of $X,W,$ and $D$ were set as missing.

#### Missing data methods and evaluation

Probabilistic bias analyses, Bayesian SM and Monte Carlo NARFCS, were implemented as described previously Based on running standard convergence checks [40] on one randomly selected dataset, Bayesian SM was applied with 50,000 iterations, of which 5,000 were burn-in iterations. Monte Carlo NARFCS was applied with 10,000 Monte Carlo steps and single imputation within each step. To assess sensitivity to the number of Monte Carlo steps and imputed datasets, we also conducted Monte Carlo NARFCS using 10,000 Monte Carlo steps with five imputations, and 5,000 Monte Carlo steps with single imputation. The number of burn-in iterations of the FCS algorithm was always set to 10. We applied Bayesian SM and Monte Carlo NARFCS with three different priors for the bias parameter: (i) vague prior $N\left(\text{0,100}\right)$, (ii) informative prior $N\left(truth,4\right)$, and (iii) very informative prior $N\left(truth,1\right)$, where $truth$ denotes the true value of the bias parameter. Note that the true value of ${\delta }^{NARFCS}$ was unknown (since it was not a parameter of either data generating model) and so we instead used an estimate of ${\delta }^{NARFCS}$ based on a simulated dataset of 50,000,000 observations.

We compared Bayesian SM and Monte Carlo NARFCS to a CCA and MI. We applied MI using FCS imputation with 10 burn-in iterations and 50 imputations, imputing the binary and continuous variables using logistic and linear regressions, respectively. (See Supplementary materials for further details on all missing data methods).

The estimand of interest was the exposure effect ${\beta }_{X}$. For the SM data generating model, the true value of ${\beta }_{X}$ was known as it was a parameter of this model, whilst for the PMM data generating model, a value for ${\beta }_{X}$ was computed by fitting the substantive analysis to a dataset of size 50,000,000 before data deletion. Performance measures of interest were bias, empirical, and model-based standard errors, and 95% confidence interval (CI) coverage of estimates of ${\beta }_{X}$. We used Stata version 17.0 [48] to generate the data. The remaining methods were conducted in R 4.1.0 [52]. Bayesian SM and Monte Carlo NARFCS were applied using high performance computing for parallel processing [53] across the simulated datasets. R package *rsimsum* (version 0.11.3) [54] was used to compute the simulation results.

### Motivating example

The motivating example for our simulation study is a previously described study where the substantive analysis of interest is a logistic regression of SARS-CoV-2 infection (0 not infected, 1 infected) on body mass index (BMI) adjusted for confounders age, sex (0 female, 1 male), university degree (0 no, 1 yes), and current smoker (0 no, 1 yes) [55]. There are three auxiliary variables: diagnosis of asthma (0 no, 1 yes), diabetes (0 no, 1 yes), and hypertension (0 no, 1 yes). This motivating example illustrates derivation of an informative prior for ${\delta }^{SM}$ and ${\delta }^{NARFCS}$. As this is an illustrative example, we have ignored other potential sources of bias (such as selection bias due to non-random participation in UK Biobank [56]), and we have only considered a small number of confounders of the outcome–exposure relationship.

#### Motivating case study

Using data from the UK Biobank study (UKB) [56], we define our target population as middle aged and elderly adults (aged 47 – 86, with close to 75% of participants aged 61 or older) resident and alive in England on 1st January 2020. Active SARS-CoV-2 infection was defined as either a positive SARS-CoV-2 PCR test (from linked Public Health England data) or COVID-19 recorded on a death certificate between 1st January 2020 and 18th May 2020 (i.e., the date mass testing became available in the UK; [57]). Testing for SARS-CoV-2 was highly restricted during this period and so data on SARS-CoV-2 infection were missing for over 98% of participants. Data on SARS-CoV-2 infection were suspected to be MNAR since testing among the majority of the UK population (i.e., non-healthcare workers) was mainly restricted to those who experienced symptoms of COVID-19 [58]. Observed factors associated with the chance of being tested in UKB included having higher BMI, being a current smoker, having a pre-existing condition (such as asthma, diabetes, or hypertension), being female, and having a university degree or higher [55].

**Fig. 2:**
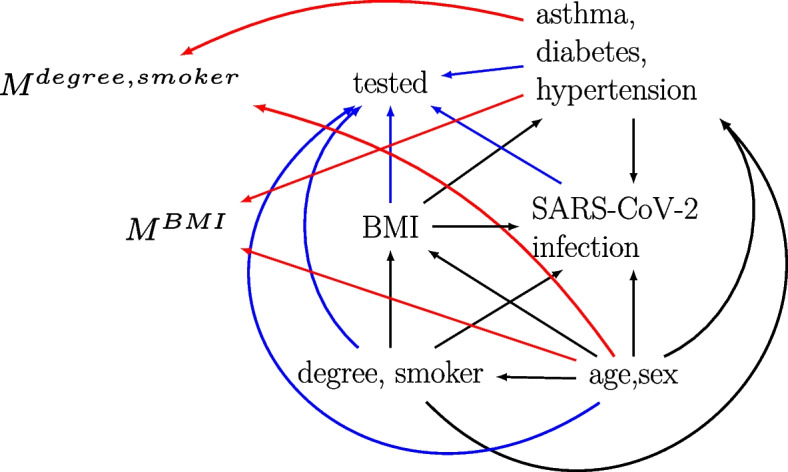
Missingness directed acyclic graph for the UK Biobank example. Black edges depict the assumed relationships in the fully observed data between the outcome (SARS-CoV-2 infection), exposure (body mass index (BMI)), confounders (age, sex, degree, and smoker), and auxiliary variables (asthma, diabetes, and hypertension). Tested, M^BMI^, and M^degree,smoker^ denote missingness indicators for the outcome, exposure, and confounders, respectively. Blue and red edges depict the missingness mechanisms of the outcome and covariates (exposure and confounders), respectively. Note, we have not included all edges between the variables

Among the 445,377 participants included in the UKB study, we excluded 24,465 (5.49%) participants who died before 2020 and 65 (0.0146%) who were not tested for SARS-CoV-2 but were diagnosed with COVID-19 post-mortem. Of the remaining 420,847 participants eligible for analysis, 405,174 (96.3%) were missing the outcome only, 10,870 (2.58%) were missing the outcome and at least one covariate (BMI, smoker, or degree), 4,610 (1.10%) had complete data and 193 (0.0459%) had an observed outcome but were missing at least one covariate (Supplementary Table 3). Confounders age and sex, and auxiliary variables asthma, diabetes, and hypertension were fully observed. Figure 2 shows the m-DAG for this motivating example based on subject-matter knowledge and our investigations of observed predictors of missingness (Supplementary tables 4 and 5). We assume the covariate data were MAR and there were no unmeasured common causes after accounting for age, sex, degree, smoker, BMI, asthma, diabetes, and hypertension.

#### Statistical analyses

We analysed the data using CCA, MI, Bayesian SM, Monte Carlo NARFCS, and a “population-based comparison group approach” where untested participants were assumed to be not infected with SARS-CoV-2 [59–61]. (See the Supplementary materials for further details). Due to convergence problems encountered when applying Bayesian SM to the full data, we restricted all analyses to the 409,784 participants with complete data on the covariates. This simplified the imputation, weighting, and bias models by reducing the number of parameters to be estimated. Given the small percentage of dropped participants (the majority of which had a missing outcome), the characteristics of the full sample and the restricted sample were virtually the same (Supplementary Table 6). In keeping with the preceding paper [55], and to improve the efficiency of MCMC sampling by reducing autocorrelation in the chains, each continuous variable (age and BMI) was standardized by subtracting its observed mean and dividing by its observed standard deviation. These standardised variables were used in all analyses. We applied MI with 50 imputed datasets, Bayesian SM using 50,000 MCMC iterations (including 5,000 burn-in iterations), and Monte Carlo NARFCS with 10,000 Monte Carlo steps and single imputation. Bayesian SM and Monte Carlo NARFCS were applied using an informative prior for ${\delta }^{SM}$ and ${\delta }^{NARFCS}$, respectively.

#### Derivation of the informative prior $\delta^{SM}$ and $\delta^{NARFCS}$

The hyperparameters of the informative priors $\normalsize p\left({\delta }^{SM}\right)\sim N\left({\mu }^{SM},{\sigma }^{SM}\right)$ and $\normalsize p\left({\delta }^{NARFCS}\right)\sim N\left({\mu }^{NARFCS},{\sigma }^{NARFCS}\right)$ were derived from published results of the REal-time Assessment of Community Transmission-2 (REACT-2) national study [62]. The REACT-2 study sent home-based SARS-CoV-2 antibody test kits to over 100,000 randomly sampled adults living in England between 20th June and 13th July 2020. Among 65–74-year-olds (similar age range to our study), SARS-CoV-2 antibody prevalence was estimated to be 3.2% [95% CI 2.8–3.6%] [62] by mid-July 2020.

Bias parameters ${\delta }^{SM}$ and ${\delta }^{NARFCS}$ are conditional parameters on the log-odds scale. So, we used an algorithm from Tompsett et al. [5] to compute approximate values of ${\delta }^{SM}$ and ${\delta }^{NARFCS}$ calibrated to marginal prevalences of SARS-CoV-2 infection. For prior $\normalsize p\left({\delta }^{NARFCS}\right)\sim N\left({\mu }^{NARFCS},{\sigma }^{NARFCS}\right)$, we set ${\mu }^{NARFCS}=-2.6$ (the value of ${\delta }^{NARFCS}$ calibrated to a prevalence of 3.2%) and set ${\sigma }^{NARFCS}={0.22}^{2}$ such that 95% of the sampled values of ${\delta }^{NARFCS}$ were expected to be between -3.0 and -2.2 (which were the values of ${\delta }^{NARFCS}$ calibrated to prevalences of approximately 2.2% and 4.2%, respectively). Note that we allowed for additional uncertainty because the prevalence of infection was unknown in our UKB study. The comparable prior for Bayesian SM was $p\left({\delta }^{SM}\right)\sim N\left(2.6, {0.22}^{2}\right)$. See Supplementary materials for further details.

## Results

### Simulation study results

**Fig. 3:**
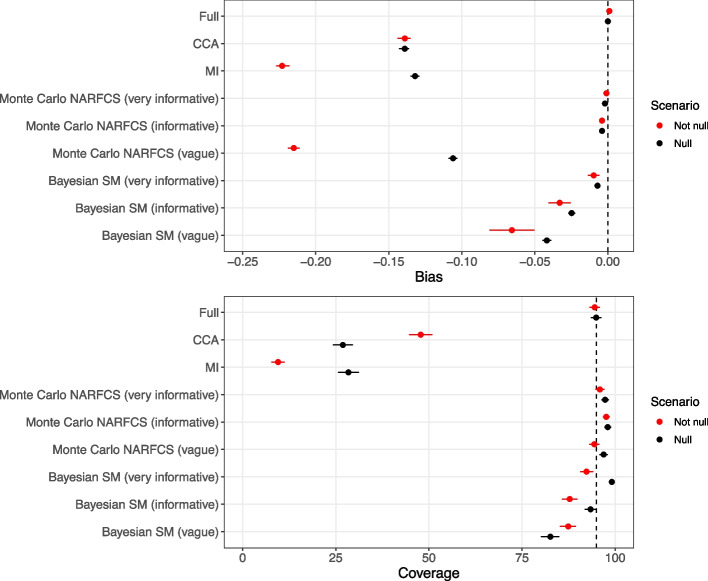
Bias and 95% confidence interval coverage of exposure effect, ${\beta }_{X}$, according to the not null (${\beta }_{X}=\text{ln}(3)$) and null (${\beta }_{X}=0$) scenarios for data generated using SM data generating model. Error bars denote 95% Monte Carlo intervals, and the vertical dashed line denotes zero bias (top) and nominal coverage (bottom). Results for Bayesian SM were based on 926–928 simulated datasets; the remaining methods were based on 1,000 simulated datasets

When there were no missing data, the full data estimate of ${\beta }_{X}$ was unbiased and CI coverage was close to the nominal level in all scenarios. Figure 3 shows the bias and coverage of estimating ${\beta }_{X}$ in the presence of missing data using different missing data methods when the true value of ${\beta }_{X}$ was $\text{ln}(3)$ and 0 and the data were generated using the SM data generating model (detailed results reported in Supplementary tables 8 and 9). There was substantial bias and severe CI under-coverage for the CCA estimates, with similar levels of bias for ${\beta }_{X}=\text{ln}(3)$ and ${\beta }_{X}=0$ but slightly higher CI coverage for ${\beta }_{X}=\text{ln}(3)$ due to wider CIs. When ${\beta }_{X}=0$, MI had broadly comparable levels of bias and CI coverage to CCA. However, when ${\beta }_{X}=\text{ln}(3)$, the bias of the MI estimates was noticeably larger than that of CCA. This was likely due to amplification of the bias (resulting from incorrect assumptions about the missingness mechanism) caused by including variables in the imputation model that were strongly predictive of $Y$ [63] (see Supplementary materials for further details).

For both the ${\beta }_{X}=\text{ln}(3)$ and ${\beta }_{X}=0$ scenarios, there was negligible bias when applying Monte Carlo NARFCS with an informative or very informative prior. Applying Monte Carlo NARFCS with a vague prior resulted in biased estimates where the level of bias was slightly lower than that of CCA for the ${\beta }_{X}=0$ scenario but higher for the ${\beta }_{X}=\text{ln}(3)$ scenario (and comparable to that of MI). In accordance with MI, the higher level of bias for the ${\beta }_{X}=\text{ln}(3)$ scenario was likely due to the auxiliary variables amplifying the bias from misspecification of the missingness mechanism. Despite the (relatively) high level of bias, CI coverage was nominal due to the imprecision of the vague prior. Very similar results were obtained when applying Monte Carlo NARFCS with 10,000 Monte Carlo steps with 5 imputations and 5,000 Monte Carlo steps with single imputation (Supplementary tables 15 and 16).

Method Bayesian SM failed to produce results for 72 to 74 simulated datasets (further details in Supplementary Sect. 3.5) whilst the other methods returned results for all 1,000 simulated datasets. Similar to Monte Carlo NARFCS, applying Bayesian SM with an informative or very informative prior resulted in minimal bias. However, compared to Monte Carlo NARFCS, Bayesian SM showed slightly higher levels of bias and inefficiency (i.e., larger empirical standard errors), leading to moderate levels of CI under-coverage. This seeming under-performance of Bayesian SM may have been due to the omitted estimates caused by nonconvergence in a small number of datasets. Unlike Monte Carlo NARFCS, Bayesian SM with a vague prior eliminated some of the bias in both the ${\beta }_{X}=\text{ln}(3)$ and ${\beta }_{X}=0$ scenarios, with bias levels at least 50% lower than those of CCA. Also, the model-based standard errors of Bayesian SM were considerably smaller than those of Monte Carlo NARFCS. A likely explanation is that some information was gained from the application of Bayes’ theorem combining the prior for ${\delta }^{SM}$ with the observed data. Supporting this claim, we note that when applied with an a priori mean of 0 for ${\delta }^{SM}$, across the simulations the mean of the posterior estimates of ${\delta }^{SM}$ was 8.83 (95% Monte Carlo interval 8.31 to 8.96) and 6.36 (95% Monte Carlo interval 6.20 to 6.52) for the ${\beta }_{X}=\text{ln}(3)$ and ${\beta }_{X}=0$ scenarios, respectively (where the true value was 7.85).

For both Bayesian SM and Monte Carlo NARFCS with (very) informative priors, there was CI overcoverage when the estimates of ${\beta }_{X}$ were unbiased (or negligibly biased). This overcoverage was likely due to generating the data using a fixed value for the bias parameter which is known to lead to CI overcoverage when applying an analysis with an informative prior centred on the true value of the parameter [64].

Similar patterns were noted on the relative performances of the methods for data generated using the PMM data generating model (Supplementary tables 18 and 19). For both data generating models and ${\beta }_{X}=\text{ln}(3)$ and ${\beta }_{X}=0$ scenarios, Bayesian SM took substantially longer to run than Monte Carlo NARFCS with Monte Carlo NARFCS taking approximately 2 days per dataset in R (approximately 1 day per dataset in Stata) and Bayesian SM taking approximately 6 days per dataset.

### Results of the motivating example

**Fig. 4:**
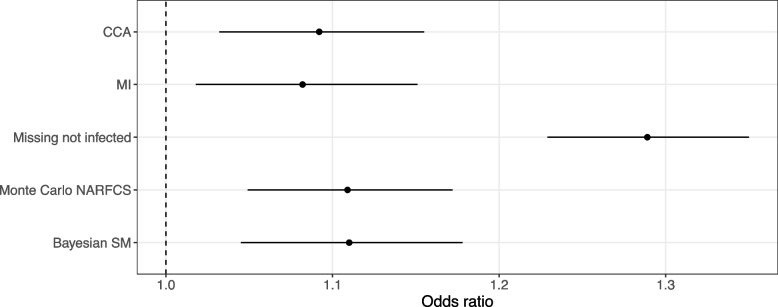
Forest plot of the results for exposure odds ratio, $exp\left\{{\beta }_{X}\right\}$, estimated by complete case analysis (CCA), multiple imputation assuming missing at random (MI), population-based comparison group approach (Missing not infected), and the probabilistic bias analyses, Monte Carlo NARFCS and Bayesian SM. Dashed line denotes the null effect

Of the 409,784 participants included in our analysis with complete covariate data, 4,610 (1.12%) were tested for SARS-CoV-2, leaving 405,174 (98.9%) with a missing outcome. Out of the 4,610 participants tested for SARS-CoV-2, 1,317 (28.6%) tested positive. Figure 4 shows the results for the exposure odds ratio (i.e., odds ratio of SARS-CoV-2 infection per standard deviation increase in BMI) estimated using CCA, MI, Bayesian SM, Monte Carlo NARFCS, and the population-based comparison group approach. All analyses suggested that participants with a higher BMI tended to be at a higher risk of SARS-CoV-2 infection. The two probabilistic bias analyses, Bayesian SM and Monte Carlo NARFCS, gave similar results with slightly higher point estimates than CCA and MI, although there was substantial overlap between the CIs of these methods. The results for the population-based comparison group approach were markedly different from those of the other methods.

The patterns in the results were consistent with our prior knowledge that untested participants tended to have a lower BMI and were less likely to have experienced symptoms of SARS-CoV-2 infection than tested participants. For example, under this missingness mechanism we expected that dropping untested participants would lead to an underestimate of the exposure odds ratio (as demonstrated by the simulation study) and setting all untested participants as “not infected” would lead to an overestimate. All analyses except CCA were based on the untested and tested participants but had similar levels of precision to that of CCA. This was unsurprising given that (i) the precision of binary outcome estimators is primarily determined by the number of cases (i.e., positive SARS-CoV-2 infections) and (ii) for our study population and study period, the prevalence of SARS-CoV-2 infection was estimated to be relatively low (3.2% [95% CI 2.8–3.6%] [62]) and so a large proportion of the untested participants were likely not infected with SARS-CoV-2. The distinct results of the population-based comparison group approach was due to the imposed extreme scenario which implied that the prevalence of infection in the study sample was only 0.32%.

## Discussion

We have illustrated the feasibility and practicality of conducting a probabilistic bias analysis to data MNAR when a large proportion of an outcome is missing under a strong MNAR mechanism. In the specific setting we considered, our simulation study demonstrated that given reasonably accurate and precise information about the bias parameter, the simpler, Monte Carlo NARFCS method performed as well as the more principled, Bayesian SM method. When very limited information was provided about the bias parameter, the Bayesian bias analysis was able to eliminate most of the bias due to data MNAR while the Monte Carlo bias analysis performed no better than the CCA and the MAR implementation of MI. We have also shown how including auxiliary variables in an imputation model can amplify bias due to data MNAR.

Monte Carlo NARFCS has three key advantages for non-specialist analysts over the Bayesian SM approach: (1) a Monte Carlo bias analysis is simpler and less daunting to implement because it does not require knowledge about Bayesian inference and specialist statistical software. (2) The bias model of Monte Carlo NARFCS uses an MNAR-extension of the popular imputation approach, FCS, which has been implemented in several software environments. (3) For our study, Monte Carlo NARFCS was less computationally demanding than Bayesian SM, resulting in substantially faster run-times. Therefore, it is encouraging that Monte Carlo NARFCS can perform as well as the more principled Bayesian SM. This is supported by previous research, which has established the robustness of FCS imputation to its theoretical weakness (that the joint distribution implied by the univariate regression models may not exist [46, 47, 65]). During the simulation study we experienced some minor technical difficulties with Bayesian SM. However, these issues can be easily resolved when applying the method in practice. For example, nonconvergence would be identified using standard Bayesian diagnostic tools and resolved by running a longer burn-in, and failure of the Bayesian sampler could be rectified by using different starting values or switching to a different Monte Carlo algorithm. In keeping with McCandless and Gustafson [16], we found that applying a Bayesian bias analysis using a vague prior for the bias parameter gained some information about the MNAR mechanism and consequently eliminated some of the bias due to missing data. This was likely due to the Bayesian process ruling out certain MNAR mechanisms (i.e., values of the bias parameter) incompatible with the observed data [16]. In contrast, since the Monte Carlo bias analysis samples directly from the prior distribution of the bias parameter, irrespective of the observed data, then applying Monte Carlo NARFCS with a vague prior performed as badly as the MAR methods. Therefore, a Bayesian bias analysis is recommended when there is limited information available about the bias parameters.

Another difference between the two probabilistic bias analyses is that the bias model of Bayesian SM is a selection model while that of Monte Carlo NARFCS is a pattern-mixture model. The advantage of the selection model framework is that it is coherent with our understanding of how the observed data arises and there is a logical separation of the parameters of interest from the bias parameters [66]. However, others have argued that the bias parameters of the pattern-mixture model are usually easier to interpret and so this framework is more convenient for conducting bias analyses [67–69]. In our applied example, the available external information was not ideally suited for the bias parameter of either the selection or pattern-mixture model. Overall, the pattern-mixture framework is credited as being more accessible and widely available [70], although the selection model framework may be preferable when the missingness mechanism is of primary interest.

Our simulation study has several limitations. First, our comparison of a Bayesian bias analysis to a Monte Carlo bias analysis also differed with respect to the bias model. However, the primary objective of our study was to illustrate an easy to apply probabilistic bias analysis (for non-specialist analysts) and to compare it to a principled approach. Second, we simulated the data using a fixed value for the bias parameter (as opposed to sampling from an appropriate prior). However, we consider the anticipated overcoverage of the probabilistic bias analyses acceptable as we focus on what Rubin terms confidence validity (i.e., intervals that cover at least nominally) rather than randomisation validity (i.e., intervals that cover exactly nominally) [71]. Third, we only explored a small number of scenarios because of the time it took to run each probabilistic bias analysis in a large data setting (typical of cohort studies). To achieve our objective of evaluating the robustness of Monte Carlo NARFCS using a small-scale simulation study, we considered an extreme setting of a large proportion of missing data with a strong MNAR mechanism.

We note that the sequential modelling approach of Bayesian SM and the FCS-type approach of Monte Carlo NARFCS can both flexibly incorporate nonlinear terms and interactions between the outcome and the predictors (i.e., covariates and auxiliary variables), and between the predictors (e.g., [34, 37, 72]). Future work should compare Bayesian SM and Monte Carlo NARFCS when the bias models include nonlinear or interaction terms.

Alternative approaches to a bias analysis are available [11]. These include (i) reference based methods used for handling missing data in randomized clinical trials (e.g., [73]), (ii) placing restrictions on the model parameters (e.g., [74]), (iii) instrumental variable(s) for missingness [6], and (iv) use of additional data (e.g., information from recontacting nonparticipants [75]).

In the extreme setting we explored, our simpler Monte Carlo bias analysis is a viable alternative to a Bayesian bias analysis provided information is available on plausible values of the bias parameter. However, when limited information is available, a Bayesian bias analysis is preferred. By illustrating two different types of probabilistic bias analyses and providing code to replicate them, we hope to encourage the increased adoption of such bias analyses in epidemiological research. Finally, in keeping with [63, 76], we caution careful consideration of the choice of auxiliary variables when applying MI where data may be MNAR.

## Supplementary Information

Supplementary Material 1.

## Data Availability

The software code to generate the simulated datasets analysed during the simulated study are available in the COVIDITY_ProbQBA repository, https://github.com/MRCIEU/COVIDITY_ProbQBA. The UK Biobank study dataset analysed during the current study is available from the UK Biobank Access Management Team (https://www.ukbiobank.ac.uk/learn-more-about-uk-biobank/contact-us) but restrictions may apply to the availability of these data, which were used under license for the current study, and so are not publicly available. All methods discussed in this paper can be implemented using the provided software code available from the COVIDITY_ProbQBA repository, https://github.com/MRCIEU/COVIDITY_ProbQBA.

## Abbreviations

BMI: Body mass index
CCA: Complete case analysis
FCS: Fully conditional specification
IPW: Inverse probability weighting
MAR: Missing at random
MCMC: Markov Chain Monte Carlo
MI: Multiple imputation
MNAR: Missing not at random
NARFCS: Not-at-random fully conditional specification
PMM: Pattern mixture model
REACT-2: REal-time Assessment of Community Transmission-2
SM: Selection model
UKB: UK Biobank study
